# Long noncoding RNA GSTM3TV2 upregulates LAT2 and OLR1 by competitively sponging let-7 to promote gemcitabine resistance in pancreatic cancer

**DOI:** 10.1186/s13045-019-0777-7

**Published:** 2019-09-12

**Authors:** Guangbing Xiong, Chang Liu, Gang Yang, Mengyu Feng, Jianwei Xu, Fangyu Zhao, Lei You, Li Zhou, Lianfang Zheng, Ya Hu, Xiaowo Wang, Taiping Zhang, Yupei Zhao

**Affiliations:** 1 Department of General Surgery, Peking Union Medical College Hospital, Chinese Academy of Medical Sciences and Peking Union Medical College, No. 1 Shuaifuyuan, Wangfujing Street, Beijing, 100730 China; 2 Department of Biliary-Pancreatic Surgery, Affiliated Tongji Hospital, Tongji Medical College, Huazhong University of Science and Technology, Wuhan, 430030 Hubei Province China; 3 MOE Key Laboratory of Bioinformatics, Bioinformatics Division and Centre for Synthetic and Systems Biology, TNLIST/Department of Automation, Tsinghua University, Haidian District, Beijing, 100084 China; 4 Department of General Surgery, Qilu Hospital, Shandong University, Jinan, 250012 Shandong Province China; 5 Department of Nuclear Medicine, Peking Union Medical College Hospital, Chinese Academy of Medical Sciences and Peking Union Medical College, Beijing, 100730 China; 6 Clinical Immunology Center, Chinese Academy of Medical Sciences, No. 1 Shuaifuyuan, Wangfujing Street, Beijing, 100730 China

**Keywords:** Pancreatic cancer, Chemoresistance, ceRNA, lncRNA, GSTM3TV2, Prognosis

## Abstract

**Background:**

Chemoresistance is one of the main causes of poor prognosis in pancreatic cancer patients. Understanding the mechanisms implicated in chemoresistance of pancreatic cancer is critical to improving patient outcomes. Recent evidences indicate that the long noncoding RNAs (lncRNAs) are involving in chemoresistance of pancreatic cancer. However, the mechanisms of lncRNAs contribute to resistance in pancreatic cancer and remain largely unknown. The objective of this study is to construct a chemoresistance-related lncRNA-associated competing endogenous RNA (ceRNA) network of pancreatic cancer and identify the key lncRNAs in regulating chemoresistance of the network.

**Methods:**

Firstly, lncRNA expression profiling of gemcitabine-resistant pancreatic cancer cells was performed to identify lncRNAs related to chemoresistance by microarray analysis. Secondly, with insights into the mechanism of ceRNA, we used a bioinformatics approach to construct a chemoresistance-related lncRNAs-associated ceRNA network. We then identified the topological key lncRNAs in the ceRNA network and demonstrated its function or mechanism in chemoresistance of pancreatic cancer using molecular biological methods. Further studies evaluated its expression to assess its potential association with survival in patients with pancreatic cancer.

**Results:**

Firstly, we demonstrated that lncRNAs were dysregulated in gemcitabine-resistant pancreatic cancer cells. We then constructed a chemoresistance-related lncRNA-associated ceRNA network and proposed that lncRNA *Homo sapiens* glutathione S-transferase mu 3, transcript variant 2 and noncoding RNA (GSTM3TV2; NCBI Reference Sequence: NR_024537.1) might act as a key ceRNA to enhance chemoresistance by upregulating L-type amino acid transporter 2 (LAT2) and oxidized low-density lipoprotein receptor 1(OLR1) in pancreatic cancer. Further studies demonstrated that GSTM3TV2, overexpressed in gemcitabine-resistant cells, enhanced the gemcitabine resistance of pancreatic cancer cells in vitro and in vivo. Mechanistically, we identified that GSTM3TV2 upregulated LAT2 and OLR1 by competitively sponging let-7 to promote gemcitabine resistance. In addition, we revealed that the expression levels of GSTM3TV2 were significantly increased in pancreatic cancer tissues and were associated with poor prognosis.

**Conclusion:**

Our results suggest that GSTM3TV2 is a crucial oncogenic regulator involved in chemoresistance and could be a new therapeutic target or prognostic marker in pancreatic cancer.

**Electronic supplementary material:**

The online version of this article (10.1186/s13045-019-0777-7) contains supplementary material, which is available to authorized users.

## Introduction

Pancreatic cancer is one of the deadliest malignancies, with an overall 5-year survival rate of 8%, and it is expected that it will become the second leading cause of cancer-related death in the US by 2030 [1, 2]. Due to a lack of diagnostic symptoms during the early disease stages, 80~85% of patients lost the opportunity to operation when diagnosed as pancreatic cancer [3, 4]. Chemotherapy, mostly gemcitabine and gemcitabine-based combinations, is indispensable in the treatment for these unresectable pancreatic cancer patients [4–6]. However, most of them suffered from a very poor prognosis with less than 1-year overall survival for chemoresistance [7, 8]. Thus, elucidating the molecular mechanism of chemoresistance is an important approach to improve the prognosis of patients with pancreatic cancer.

Long noncoding RNAs (lncRNAs) are a class of largely functional transcripts longer than 200 nucleotides that have been shown to involve in the pathogenesis of cancer by acting as oncogenes or tumour suppressor genes, in regulating of cell cycle, survival, apoptosis, angiogenesis, pluripotency, invasion, metastasis, etc. [9–12]. Notably, some lncRNAs constitute critical contributors to various known or unknown mechanisms of chemoresistance and are important determinants of the efficacy of anticancer therapies in cancer, including pancreatic cancer [9, 13, 14]. Thus, a better understanding of the biology of lncRNAs might uncover mechanisms of therapeutic strategies for pancreatic cancer. However, only a minor fraction of lncRNAs related to drug resistance have been functionally annotated in pancreatic cancer, and the knowledge regarding mechanisms is also limited [9]. Recent studies have demonstrated that certain lncRNAs can act as competing endogenous RNA (ceRNAs) or miRNA “sponges” to modulate chemotherapy sensitivity in pancreatic cancer, such as linc-ROR [15], GAS5 [16, 17] and linc-DYNC2H1-4 [18]. Additionally, construction and analysis of dysregulated lncRNA-mediated ceRNA network have been indicated to be a feasible way to understand the regulatory mechanisms in the pathogenesis of pancreatic cancer and provide novel lncRNAs as candidate diagnostic biomarkers or potential therapeutic targets [19–21]. Construction of the chemoresistance-related lncRNA-associated ceRNA network and identified key regulator of the ceRNA network in pancreatic cancer has not yet been perceived. Therefore, identifying a ceRNA network related to chemoresistance and investigating its underlying mechanism may provide potential therapeutic targets for improving the prognosis of pancreatic cancer.

The current study was aimed to construct a chemoresistance-related lncRNA-associated ceRNA network of pancreatic cancer and demonstrate key regulator of chemoresistance in the ceRNA network. We found that lncRNA *Homo sapiens* glutathione S-transferase mu 3, transcript variant 2, noncoding RNA (GSTM3TV2; NCBI Reference Sequence: NR_024537.1) and overexpressing in gemcitabine-resistant pancreatic cancer cells, acted as a key regulator of chemoresistance in the ceRNA network. We then investigated that GSTM3TV2 could promote pancreatic cancer gemcitabine resistance by upregulating L-type amino acid transporter 2 (LAT2) and oxidized low-density lipoprotein receptor 1(OLR1) though competitively sponging let-7. In addition, we detected the GSTM3TV2 expression was significantly upregulated in pancreatic cancer tissues, and high expression of GSTM3TV2 had a worse prognosis. Taken together, these results indicate that GSTM3TV2 could be a new therapeutic target and prognostic marker in pancreatic cancer.

## Materials and methods

### Patients and specimens

Pancreatic adenocarcinoma patient tissue samples were obtained from Peking Union Medical College Hospital (Beijing, China) and patients were enrolled based on a confirmed histological diagnosis. A total of 180 formalin-fixed, paraffin-embedded pancreatic adenocarcinoma specimens and matched tumour-adjacent tissues were used to construct tissue microarrays to detect the expression of the lncRNA GSTM3TV2. None of the patients received neoadjuvant therapy before surgical resection. The project protocol was approved by the Ethics Committees of the Peking Union Medical College Hospital, and written informed consent was obtained from all patients enrolled in this study.

### Cell lines and culture

AsPC-1 and MIAPaCa-2 pancreatic ductal adenocarcinoma (PDAC) cells (a generous gift from Professor Helmut Freiss at Heidelberg University, Germany) were cultured in a humidified incubator containing 5% CO_2_ at 37 °C in either RPMI 1640 medium or Dulbecco’s modified Eagle’s medium (DMEM, HyClone, Thermo Fisher Scientific Inc., Waltham, MA) supplemented with 10% foetal bovine serum (FBS; HyClone). The 293A cell line was purchased from Cell Resource Centre (IBMS, CAMS/PUMC) and cultured in RPMI 1640 containing 10% FBS. The gemcitabine-resistant cell lines AsPC-1/GR and MIAPaCa-2/GR were generated by intermittently increasing the drug concentration. The initial concentration used was the half maximal inhibitory concentration (IC50; AsPC-1, 2.711 μmol/L; MIAPaCa-2, 7.413 μmol/L). The drug concentration was increased exponentially up to 1000 μg/mL over a period of 9 months. The IC50 values for the AsPC-1/GR and MIAPaCa-2/GR cells were 668.860 μmol/L and 477.485 μmol/L. Gemcitabine (750 ng/mL) was included in the medium to maintain the resistant phenotype and removed 1 month before the cells were subjected to experiments.

### Microarray analysis

Total RNA was extracted from AsPC-1 and AsPC-1/GR cells using TRIzol RNA isolation reagent (Invitrogen, Carlsbad, CA, USA) according to the manufacturer’s protocol. RNA quality and quantity were assessed using capillary electrophoresis with Fragment Analyzer and Standard/High Sensitivity RNA Analysis kits (Advanced Analytical Technologies, Ames, IA). To identify the lncRNA profiles associated with chemoresistance of pancreatic cancer, an Affymetrix GeneChip Human Transcriptome Array 2.0 (Affymetrix) was used according to the manufacturer’s protocol. Biotinylated complementary DNA (cDNA) was prepared from 500 ng of total RNA. After labelling and hybridization, the GeneChips were washed and stained using an Affymetrix Fluidics Station 450 and then scanned with an Affymetrix GeneChip Scanner 3000 7G. The data were analysed with a Robust Multichip Analysis algorithm using the Affymetrix default analysis settings with global scaling as the normalization method. To determine the significance of the differences and the false discovery rate (FDR), thresholds of *P* < 0.05 and FDR < 0.05 were used. Gene expression fold changes of either > 2 or < 0.5 were set as the default filter criteria for identifying significant differentially expressed genes.

### RNA reverse transcription and quantitative real-time PCR

To synthesize cDNA, total RNA was reverse transcribed using a PrimeScript RT Reagent Kit (Takara, Japan) and a miRNA qPCR Quantitation Kit (GenePharma, Shanghai, China) according to the manufacturer’s instructions. Quantitative real-time RT-PCR was performed using a StepOnePlus™ System (Applied Biosystems, Foster City, CA, USA) with SYBR Green Master Mix (Takara, Japan). Glyceraldehyde-3-phosphate dehydrogenase (GAPDH) and U6 snRNA were used as endogenous controls for mRNA and miRNA, respectively. All samples were normalized to internal controls, and the fold changes were calculated using a relative quantification method (2^−ΔΔCt^). Real-time PCR reactions were performed in triplicate. The primer sequences are listed in Additional file 1: Supplemental Information.

### Western blot analysis

Cells seeded in six-well plates were harvested 48 h after transfection and lysed with RIPA buffer (Applygen, Beijing). Total cell lysates (100 μg) were separated by sodium dodecyl sulphate polyacrylamide gel electrophoresis and transferred to a polyvinylidene difluoride membrane (Millipore, Billerica, MA). After the membranes were blocked in 5% skim milk at room temperature for 1 h, they were incubated with primary antibodies overnight at 4 °C. The membranes were then probed with horseradish peroxidase-conjugated secondary antibodies at room temperature for 1 h and visualized using an enhanced chemiluminescence detection system (ECL Plus Western Blotting Detection System; Amersham Biosciences, Foster City, CA, USA). Band intensities were quantified using Image-Pro Plus 6.0 software (Media Cybernetics, USA). The primary antibodies used are listed in Additional file 1: Supplemental Information.

### Vector construction

Complementary DNA encoding GSTM3TV2 was synthesized and subcloned into the pcDNA3.1(+) vector (Invitrogen) according to the manufacturer’s instructions. The pcDNA3.1-GSTM3TV2 construct containing point mutations at the putative let-7 binding sites was synthesized by Imagen Therapeutics (Beijing, China) and named pcDNA3.1-GSTM3TV2-Mut. The pSL-MS2-12X (Addgene) was double-digested with BamH I and Xba I, and the MS2-12X fragment was subcloned into the pcDNA3.1, pcDNA3.1-GSTM3TV2 and pcDNA3.1-GSTM3TV2-Mut vectors to generate pcDNA3.1-MS2, pcDNA3.1-MS2-GSTM3TV2 and pcDNA3.1-MS2-GSTM3TV2-Mut, respectively. The let-7 binding region in either lncRNA-GSTM3TV2 or lncRNA-GSTM3TV2-Mut was amplified using PCR and subcloned into the pmirGLO vector (Promega, Madison, WI, USA) for use in a luciferase reporter assay.

### Cell transfection

Transfections were performed using Lipofectamine 3000 and OPTI-MEM (Invitrogen) according to the manufacturer’s instructions. The let-7 miRNA mimics hsa-let-7d-5p, hsa-let-7f-5p and hsa-let-7 g-5p; miRNA negative control; and siGSTM3TV2 and siNC were purchased from RiboBio (Guangzhou, China) and introduced into cells at a final concentration of 50 nM. The transfected cells were harvested at 48 h after transfection. The sequences for siRNA are listed in Additional file 1: Supplemental Information.

### Growth inhibition assay

The growth inhibition assay was performed using Cell Counting Kit-8 reagent (Dojindo, Tokyo, Japan) according to the manufacturer’s protocol. To measure the effects of GSTM3TV2 on chemosensitivity, cells were seeded in six-well plates, transfected for 24 h and trypsinized and reseeded in 96-well plates (4000 cells/well). Then, the cells were incubated with different concentrations of gemcitabine (Eli Lilly and Company, USA), for an additional 48 h. The OD450 was measured after adding the CCK-8 reagent (10 μL/well) for an additional 2.5 h at 37 °C, and the growth inhibition rate was calculated as follows: $\mathrm{inhibition}\ \mathrm{rate}=1-\frac{{\mathrm{OD}}_{\mathrm{Gem}}-{\mathrm{OD}}_{\mathrm{blank}}}{{\mathrm{OD}}_{\mathrm{control}}-{\mathrm{OD}}_{\mathrm{blank}}}$. The OD450 value of the cell with different concentrations of gemcitabine marked as OD_Gem_, the OD450 value of cells without gemcitabine treatment marked as OD_control_ and the OD450 value of culture medium was marked as OD_blank_

### Apoptosis assay

Pancreatic cancer cells seeded into six-well plates were transfected as indicated for 24 h. To determine the chemosensitivity of these cells, gemcitabine was added for 48 h, after which the cells were collected and resuspended in binding buffer. The cells were then stained with annexin V-FITC and PI (Beyotime, China) according to the manufacturer’s instructions and analysed by flow cytometry (FACScan; BD Biosciences, USA).

### Luciferase reporter assays

The pmirGLO dual-luciferase miRNA target expression vector (Promega, E1330) was used to assess let-7 regulation of putative miRNA target sites. Vectors and either let-7 mimics or a mimic control were co-transfected into 293A cells in 12-well plates (1 × 10^5^ cells/well) using Lipofectamine 3000 reagent. After 48 h, the luciferase activities were evaluated using a Dual-Luciferase Reporter Assay System (Promega) according to the manufacturer’s guidelines. Renilla luciferase (hRlucneo) served as the control reporter for normalization.

### RNA immunoprecipitation

AsPC-1 cells were co-transfected with pMS2-GFP (Addgene) and pcDNA3.1-MS2, pcDNA3.1-MS2-GSTM3TV2 or pcDNA3.1-MS2-GSTM3TV2-Mut. After 48 h, cells were subjected to RNA immunoprecipitation (RIP) experiments using a GFP antibody (Roche, Mannheim, Germany) and an EZ-Magna RIPTM RNA-Binding Protein Immunoprecipitation Kit (Millipore, Catalogue No.17-701, Bedford, MA, USA) according to the manufacturer’s instructions [22].

### In situ hybridization

A locked nucleic acid (LNA) probe with complementarity to a section of GSTM3TV2 (5Dig_N/ATGCCACAGTGAACATCTTAGT/3Dig_Ncustom LNA detection probe) (Exiqon, Vedbaek, Denmark) was used to detect GSTM3TV2 expression in tissues. In situ hybridization (ISH) was performed as previously described [23, 24]. Slides were scored according to the staining intensity and number of positive cells. Scoring for staining intensity was as follows: none (0), weak staining (1), intermediate staining (2) and strong staining (3). Scoring for the percentage of positive cells was as follows: absent (0), 1–24% positive cells (1), 25–49% (2), 50–74% (3) and 75–100% (4). The final score was calculated by multiplying the scores for intensity and percentage, ranging from 0 to 12. GSTM3TV2 expression was considered low if the final score was less than 4 points and high if the final score was 4 or more points.

### Animal experiments

AsPC-1 cells stably transfected with either GSTM3TV2-retroviral vectors or control retroviral vectors were subcutaneously injected into the right back of 6-week-old female BALB/c mice (Shanghai, Chinese Academy of Sciences, China) (5 × 10^6^ cells in 250 μl of PBS per mouse). Each experimental group included five mice. Tumour size was measured twice a week using calliper measurements of two perpendicular diameters of the implants. Tumour volume (cubic millimetre) was calculated based on the formula volume (mm3) = 1/2 × length × width^2^. To determine the effects of GSTM3TV2 on chemosensitivity in vivo, the animals were intraperitoneally injected with gemcitabine (25 mg/kg, twice weekly) beginning 1 week after inoculation for 4 weeks. The tumour-bearing mice were euthanized after 30 days of drug treatment.

### Statistical analysis

Statistical analysis and graph representations were performed using SPSS v.13.0 software (SPSS Inc., Chicago, IL) and GraphPad Prism 5 Software (GraphPad, San Diego, CA), respectively. Measurement data are presented as the mean ± standard deviation (SD) and were compared using either Student’s *t* test or the Mann-Whitney *U* test. Categorical data were compared using either the Pearson *χ*^2^ test or Fisher’s exact test. The Kaplan-Meier method and Cox regression were used for univariate and multivariate survival analyses. A value of *P* < 0.05 was considered statistically significant.

## Results

### Constructing a chemoresistance-related lncRNA-associated ceRNA network of pancreatic cancer

**Fig. 1:**
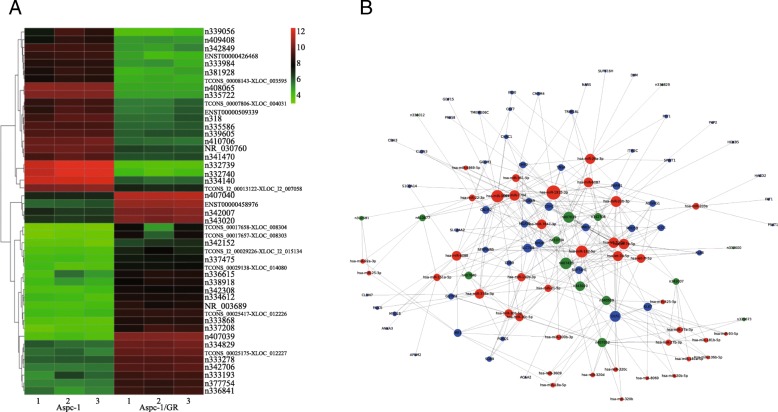
Construction of a chemoresistance-related lncRNA-associated ceRNA network of pancreatic cancer by bioinformatics analysis. **a** The hierarchical clustering of dysregulated lncRNAs expression profiling between gemcitabine-resistant AsPC-1/GR cells and AsPC-1 cells. The lncRNA GSTM3TV2 (Probe ID: n407039) was upregulated in AsPC-1/GR cells. **b** The specific chemoresistance-related lncRNA-associated ceRNA network of pancreatic cancer was constructed by bioinformatics method. LncRNA GSTM3TV2 was the predicted key nodes in ceRNA network

To construct a specific chemoresistance-related lncRNA-associated ceRNA network of pancreatic cancer, we firstly performed microarray analysis to screen lncRNAs, mRNAs and miRNAs potentially involved in gemcitabine resistance by using gemcitabine-resistant pancreatic cancer cell line AsPC-1/GR. We then identified 724 lncRNAs (fold change > 2) in AsPC-1/GR cells compared to the AsPC-1 cells, including 339 upregulated lncRNAs and 385 downregulated lncRNAs (Fig. 1a, Additional file 2: Supplementary Material 1). Concurrently, 1483 mRNAs (> 2-fold change) and 214 miRNAs (> 2.0-fold change) displayed differential expression (Additional file 3: Figure S1A–B, Additional file 4: Supplementary Material 2, Additional file 5: Supplementary Material 3). Secondly, we utilized the paired dysregulated lncRNA, miRNA and mRNA expression profiles to construct the specific chemoresistance-related lncRNA-associated ceRNA network by bioinformatics method. Among the ceRNA network, we focused on lncRNAs as the key topological node for mediating the chemoresistance of pancreatic cancer (Fig. 1b). Then, we revealed that the lncRNAs n407039 (*Homo sapiens* glutathione S-transferase mu 3, transcript variant 2, noncoding RNA, GSTM3TV2; NCBI Reference Sequence: NR_024537.1) (Probe ID: n407039) and n407040 (NCBI Reference Sequence: NR_024538.1) might be the predicted key nodes for regulating chemoresistance in the ceRNA network. Further analysis demonstrated that GSTM3TV2 and several other genes, including GLO1, KLK6, LAT2, OLR1 and PARM1, share the same let-7 family miRNAs via ceRNA-based crosstalk for modulating chemoresistance.

### GSTM3TV2 is overexpressed in gemcitabine-resistant pancreatic cancer cells

**Fig. 2:**
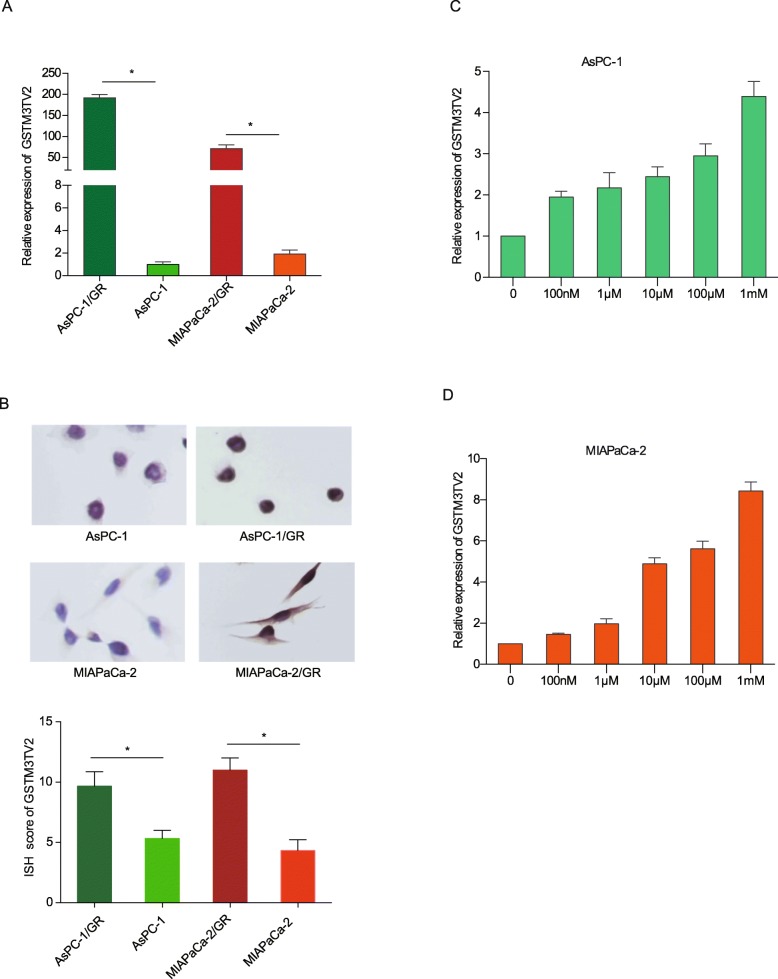
GSTM3TV2 is overexpressed in gemcitabine-resistant pancreatic cancer cells. **a** GSTM3TV2 was upregulated in gemcitabine-resistant cells AsPC-1/GR and MIAPaCa-2/GR when validated by quantitative reverse-transcription polymerase chain reaction (qRT-PCR). **b** In situ hybridization (ISH) analysis showed GSTM3TV2 was overexpressed in gemcitabine-resistant cells AsPC-1/GR and MIAPaCa-2/GR and localized in the cytoplasm. **c**, **d** GSTM3TV2 expression was increased when AsPC-1 (**c**) and MIAPaCa-2 (**d**) cells stimulated with different concentrations of gemcitabine for 48 h. The data are presented as the mean ± SD (Student’s *t* test; asterisk indicates *P* < 0.05)

We then detected GSTM3TV2 expression level in pancreatic cancer cell lines based on the microarray analysis. Quantitative RT-PCR showed that the expression level of GSTM3TV2 in gemcitabine-resistant pancreatic cancer cells AsPC-1/GR and MIAPaCa-2/GR were significantly increased than in AsPC-1 and MIAPaCa-2 cells (Fig. 2a). In situ hybridization (ISH) staining further demonstrated that GSTM3TV2 was upregulated in AsPC-1/GR and MIAPaCa-2/GR cells compared with that in AsPC-1(*P* < 0.05) and MIAPaCa-2 cells (*P* < 0.05) (Fig. 2b). And the GSTM3TV2 was mainly distributed in the cytoplasm (Fig. 2b). Moreover, GSTM3TV2 expression was increased when AsPC-1 and MIAPaCa-2 cells were stimulated with different concentrations of gemcitabine for 48 h (Fig. 2c, d). Thus, these results suggested that GSTM3TV2 was upregulated and might be involved in the development of chemoresistance in pancreatic cancer.

### GSTM3TV2 enhances gemcitabine resistance of pancreatic cancer in vitro and in vivo

**Fig. 3:**
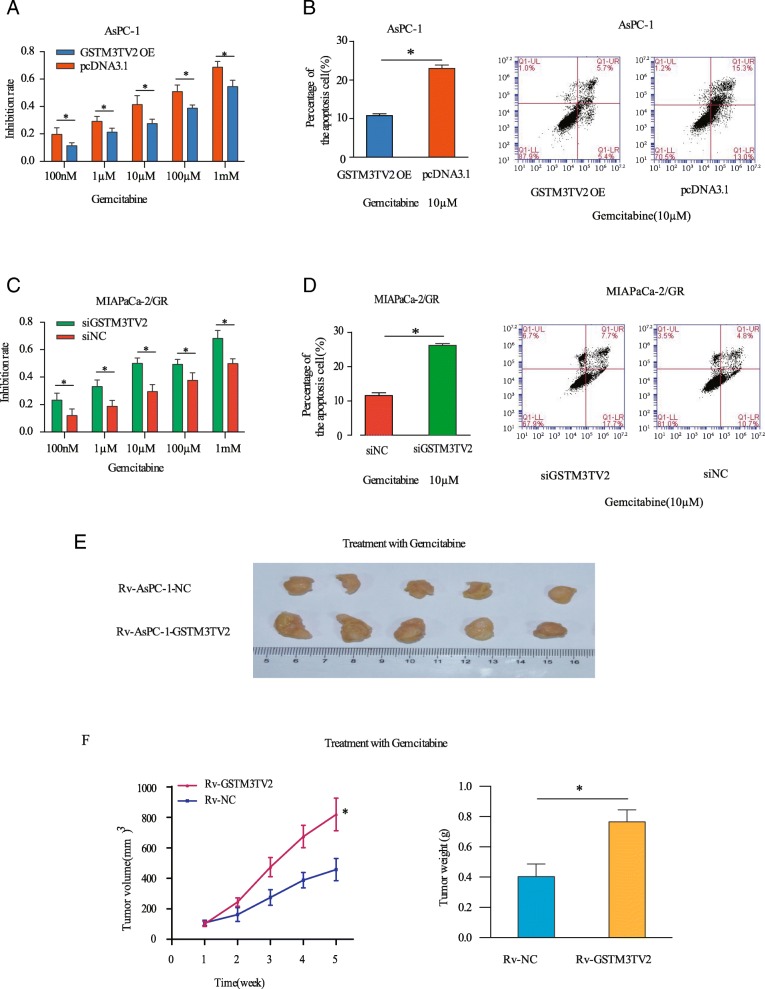
GSTM3TV2 enhances gemcitabine resistance in vivo and in vitro. **a** Overexpression of GSTM3TV2 in AsPC-1 cells decreased the inhibitory effects of gemcitabine as determined by detecting cell viability. **b** Overexpression of GSTM3TV2 in AsPC-1 cells decreased gemcitabine-induced cell death as determined by detecting apoptosis rates. **c** Knockdown GSTM3TV2 in gemcitabine-resistant MIAPaCa-2/GR cells increased chemosensitivity as determined by detecting cell viability. **d** Knockdown GSTM3TV2 in gemcitabine-resistant MIAPaCa-2/GR cells increased gemcitabine-induced cell death determined by detecting apoptosis rates. **e** Total number of tumours resected from mice 28 days treated with gemcitabine of AsPC-1 cells stable expression with GSTM3TV2 (Rv-AsPC-1-GSTM3TV2) or NC (Rv-AsPC-1-NC). **f** The tumourigenesis capability of Rv-AsPC-1-GSTM3TV2 cells and Rv-AsPC-1-NC cells were examined in vivo by measuring the tumour volume and tumour weight. The data are presented as the mean ± SD (Student’s *t* test; asterisk indicates *P* < 0.05)

To ascertain the role of GSTM3TV2 in chemoresistance in vitro, we employed a gain- or loss-of-function strategy to overexpress or knock down GSTM3TV2 in pancreatic cancer cells (Additional file 6: Figure S2A). Following the overexpression of GSTM3TV2 in AsPC-1 and MIAPaCa-2 cells, CCK-8 assay results showed a decrease in the inhibitory effects of gemcitabine compared with that in cells transfected with empty vector (Fig. 3a, Additional file 6: Figure S2B). Furthermore, the measurement of apoptosis using flow cytometry analysis revealed that cells overexpressing GSTM3TV2 exhibited a lower rate of apoptosis when incubated with gemcitabine for 48 h (Fig. 3b, Additional file 6: Figure S2C). By contrast, AsPC-1/GR and MIAPaCa-2/GR cells transfected with siGSTM3TV2 were more sensitive to gemcitabine than cells transfected with siNC (Fig. 3c, d; Additional file 6: Figure S2D–E). To further determine the effects of GSTM3TV2 on chemosensitivity in vivo, we established AsPC-1 cell lines that stably overexpressed GSTM3TV2 (Additional file 6: Figure S2F) and subcutaneously injected these cells into nude mice treated with gemcitabine (Fig. 3e). The in vivo results revealed that in mice treated with gemcitabine, the tumours generated from Rv-AsPC-1-GSTM3TV2 cells grew significantly faster than the control cells (Fig. 3f). In addition, the tumour weight was significantly increased when pancreatic cancer cells overexpressed GSTM3TV2. Thus, these results suggest that GSTM3TV2 decreases gemcitabine-induced cytotoxicity in vitro and in vivo.

### GSTM3TV2 acts as a ceRNA via sponging let-7

**Fig. 4:**
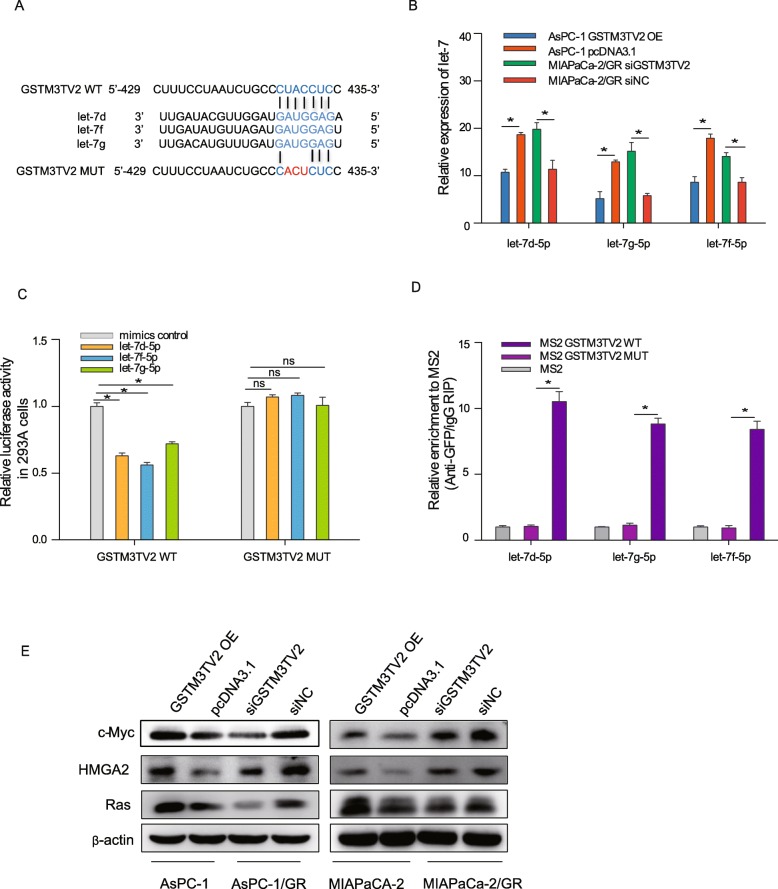
GSTM3TV2 acts as a ceRNA via sponging let-7. **a** Bioinformatics analysis predicted let-7 binding sites (position: 429~435) on GSTM3TV2. Mutations were generated at the predicted let-7 binding sites on the GSTM3TV2 sequence. **b** Relative expression levels of let-7 were significantly decreased when GSTM3TV2 was overexpressed in AsPC-1 cells; whereas, let-7 was upregulated when GSTM3TV2 was knocked down in gemcitabine-resistant MIAPaCa-2/GR cells. **c** Luciferase activity in 293A cells co-transfected with let-7 mimics and luciferase reporters containing wild-type or mutant GSTM3TV2. The luciferase activities of GSTM3TV2 WT reporter vector were reduced when transfected with let-7 mimics; whereas, let-7 had no effects on luciferase activities of GSTM3TV2 MUT reporter vector. The data are presented as the relative ratio of firefly luciferase activity to Renilla luciferase activity. **d** RNA immunoprecipitation (RIP) assay followed by miRNA qRT-PCR showed that the RIP of GSTM3TV2 in AsPC-1 cells was significantly enriched with let-7 compared to the RIP of IgG, empty vector (MS2) and GSTM3TV2 with mutations at the let-7 targeting sites. **e** Western blot assays showed that GSTM3TV2 regulates the expression of the endogenous let-7 targets c-Myc, HMGA2 and Ras in pancreatic cells as determined using loss- or gain-of-function strategies with GSTM3TV2. The data are presented as the mean ± SD (Student’s *t* test; asterisk indicates *P* < 0.05)

As bioinformatics method previously predicted that GSTM3TV2 might function as a ceRNA to sponge the let-7 family (Fig. 4a), we first examined let-7 expression that was upregulated in MIAPaCa-2/GR cells with GSTM3TV2 knockdown and downregulated in GSTM3TV2-overexpressing AsPC-1 cells (Fig. 4b). These results suggested that GSTM3TV2 may exert an impact on deregulation of let-7. Next, we constructed luciferase reporter plasmids containing the GSTM3TV2 coding sequences containing either wild-type (WT) or mutated let-7 binding sites to identify target effectors. We found that the let-7d-5p, let-7f-5p and let-7 g-5p mimics reduced the luciferase activities of the WT reporter vector, indicating that the let-7 family of miRNAs target GSTM3TV2 in 293A cells (Fig. 4c). We also performed an RNA immunoprecipitation (RIP) assay to pull down endogenous miRNAs associated with GSTM3TV2 and used qRT-PCR to show that the RIP of GSTM3TV2 in AsPC-1 cells was significantly enriched with let-7d-5p, let-7f-5p and let-7 g-5p compared to the RIP of IgG, empty vector (MS2) and GSTM3TV2 with mutations at the let-7-targeting sites (Fig. 4d). Moreover, western blot analysis showed that the expression levels of endogenous let-7 targets c-Myc, HMGA2 and Ras were increased when GSTM3TV2 overexpressing in AsPC-1 and MIAPaCa-2 cells, and decreased upon GSTM3TV2 inhibition in AsPC-1/GR and MIAPaCa-2/GR cells (Fig. 4e), which suggested that the regulation of c-Myc, HMGA2 and Ras by GSTM3TV2 was dependent on let-7-specific binding. Taken together, all these data suggest that GSTM3TV2 physically associates with let-7 and functions as a ceRNA for let-7.

### GSTM3TV2 upregulates LAT2 and OLR1 expression

**Fig. 5:**
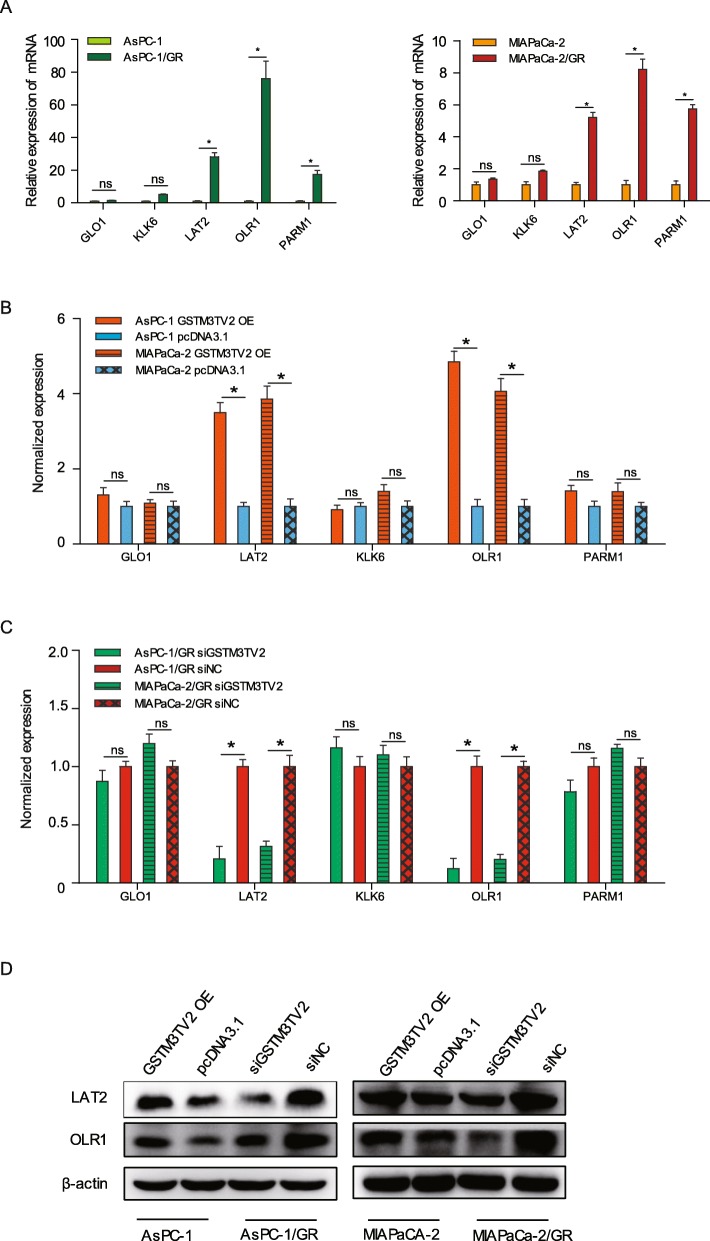
GSTM3TV2 upregulates LAT2 and OLR1 expression. **a** The expression levels of LAT2, OLR1 and PARM1 were significantly upregulated in gemcitabine-resistant AsPC-1/GR and MIAPaCa-2/GR cells than in AsPC-1 and MIAPaCa-2 cells as detected by qRT-PCR analysis. **b** The expression levels of LAT2 and OLR1 were significantly increased when GSTM3TV2 overexpresses in AsPC-1 and MIAPaCa-2 cells as detected by qRT-PCR analysis. **c** The expression levels of LAT2 and OLR1 were significantly decreased when GSTM3TV2 knock down in AsPC-1/GR and MIAPaCa-2/GR cells as detected by qRT-PCR analysis. **d** Western blot analysis shown that LAT2 and OLR1 were upregulated when GSTM3TV2 overexpresses in AsPC-1 and MIAPaCa-2 cells; whereas, LAT2 and OLR1 were downregulated when GSTM3TV2 knock down in AsPC-1/GR and MIAPaCa-2/GR cells. The data are presented as the mean ± SD (Student’s *t* test; asterisk indicates *P* < 0.05)

The above ceRNA networks of chemoresistance indicated that GSTM3TV2 could modulate the expression of GLO1, KLK6, LAT2, OLR1 and PARM1 by sponging let-7. Thus, we first validated the expression of GLO1, KLK6, LAT2, OLR1 and PARM1 by using qRT-PCR, which showed that the expression level of LAT2, OLR1 and PARM1 were significantly upregulated in AsPC-1/GR and MIAPaCa-2/GR cells than in AsPC-1 and MIAPaCa-2 cells (Fig. 5a). Then, we observed that the mRNA expression levels of LAT2 and OLR1 were significantly increased when GSTM3TV2 overexpresses in AsPC-1 and MIAPaCa-2 cells (Fig. 5b), and decreased upon GSTM3TV2 inhibition in AsPC-1/GR and MIAPaCa-2/GR (Fig. 5c). In addition, the expression of LAT2 and OLR1 at the protein level was also positively regulated by GSTM3TV2 as determined using western blot analysis (Fig. 5d). Taken together, these results suggested that GSTM3TV2 could upregulate LAT2 and OLR1 expression.

### LAT2 and OLR1 are the direct targets of let-7

**Fig. 6:**
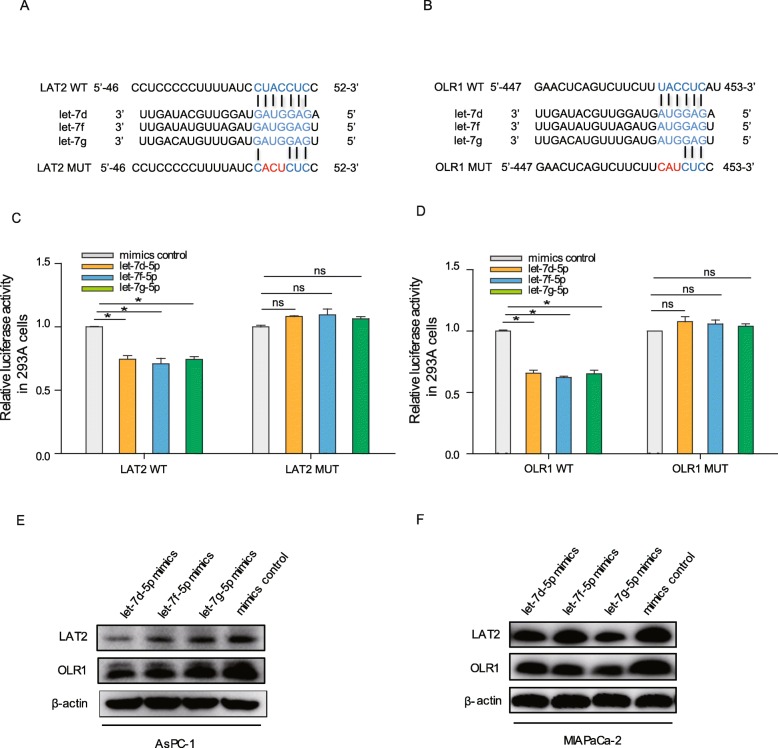
LAT2 and OLR1 are the direct targets of let-7. **a** Target scan predicted the let-7 binding sites of 3′-UTR of LAT2. Mutation was generated on the 3′-UTR of LAT2 sequence for the seed region of let-7. **b** Target scan predicted the let-7 binding sites of 3′-UTR of OLR1. Mutation was generated on the 3′-UTR of OLR1 sequence for the seed region of let-7. **c** The luciferase activities of 3′-UTR of LAT2 reporter vector were reduced when co-transfected with let-7 mimics; whereas, let-7 had no effects on luciferase activities of 3′-UTR of LAT2 reporter vector. **d** The luciferase activities of 3′-UTR of OLR1 reporter vector were reduced when co-transfected with let-7 mimics; whereas, let-7 had no effects on luciferase activities of 3′-UTR of OLR1 reporter vector. **e**, **f** Western blot analysis showed that the expression levels of LAT2 and OLR1 were downregulated when let-7 overexpresses in AsPC-1 and MIAPaCa-2 cells. The data are presented as the mean ± SD (Student’s *t* test; asterisk indicates *P* < 0.05)

To ascertain whether the above-observed effects that GSTM3TV2 increased the LAT2 and OLR1 expression depended on the regulation of the let-7, we then performed reporter assays to confirm the 3′ untranslated regions (3′-UTR) of LAT2 and OLR1 binding in predicted sites of let-7 (Fig. 6a, b). We found that the luciferase activity was significantly decreased when let-7 was ectopically expressed in cells transfected with pmirGLO-LAT2-wt or pmirGLO-OLR1-wt, compared with cells transfected with pmirGLO-LAT2-mut or pmirGLO-OLR1-mut vector (Fig. 6c, d). Then, western blot analysis showed that the expression levels of LAT2 and OLR1 were decreased when let-7 overexpresses in AsPC-1 and MIAPaCa-2 cells (Fig. 6e, f). Thus, these results indicated that GSTM3TV2 upregulated LAT2 and OLR1 expression by competitively sponging let-7.

### GSTM3TV2 promotes gemcitabine resistance dependent on the expression of LAT2 and OLR1

**Fig. 7:**
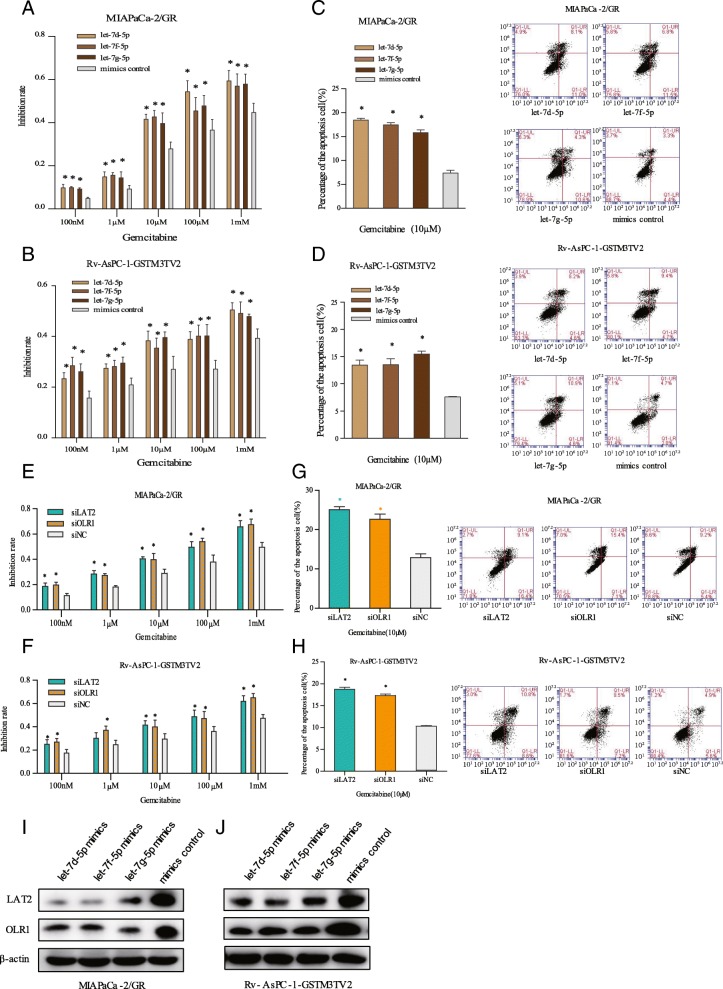
GSTM3TV2 promotes gemcitabine resistance dependent on the expression of LAT2 and OLR1. **a** Overexpression of let-7 in MIAPaCa-2/GR cells increased the inhibitory effects of gemcitabine as determined by detecting cell viability. **b** Overexpression of let-7 in Rv-AsPC-1-GSTM3TV2 cells increased the inhibitory effects of gemcitabine as determined by detecting cell viability. **c** Overexpression of let-7 in MIAPaCa-2/GR cells increased gemcitabine-induced cell death as determined by detecting apoptosis rates. **d** Overexpression of let-7 in Rv-AsPC-1-GSTM3TV2 cells increased gemcitabine-induced cell death as determined by detecting apoptosis rates. **e** Knockdown LAT2 and OLR1 in MIAPaCa-2/GR cells increased the inhibitory effects of gemcitabine as determined by detecting cell viability. **f** Knockdown LAT2 and OLR1 in MIAPaCa-2/GR cells increased gemcitabine-induced cell death as determined by detecting apoptosis rates. **g** Knockdown LAT2 and OLR1 in Rv-AsPC-1-GSTM3TV2 cells increased the inhibitory effects of gemcitabine as determined by detecting cell viability. **h** Knockdown LAT2 and OLR1 in Rv-AsPC-1-GSTM3TV2 cells increased gemcitabine-induced cell death as determined by detecting apoptosis rates. **i**, **j** Western blot analysis showed that the expression levels of LAT2 and OLR1 were downregulated when let-7 overexpresses in MIAPaCa-2/GR and Rv-AsPC-1-GSTM3TV2 cells

We then validated the dependence of GSTM3TV2-enhanced gemcitabine resistance on LAT2 and OLR1. Firstly, we performed CCK-8 assay to detect the chemoresistance of GSTM3TV2 medicated in pancreatic cancer cells by sponging let-7. Data showed that MIAPaCa-2/GR and Rv-AsPC-1-GSTM3TV2 cells transfected with the let-7 mimics were more sensitive to gemcitabine than cells transfected with mimic controls (Fig. 7a, b). For further confirmation, flow cytometry analysis revealed that cells overexpressing let-7 exhibited a higher rate of apoptosis when incubated with gemcitabine for 48 h (Fig. 7c, d). Thus, the results demonstrated that GSTM3TV2-enhanced gemcitabine resistance is dependent on sponging of let-7. Next, to validate whether GSTM3TV2 modulated LAT2 and OLR1 to promote chemoresistance in pancreatic cancer, we constructed the specific siRNAs targeting LAT2 and OLR1 (siLAT2 and siOLR1, respectively) to silence the expression of LAT2 and OLR1 in MIAPaCa-2/GR and Rv-AsPC-1-GSTM3TV2 cells (Additional file 6: Figure S2G, H). Compared with cells transfected with siNC, cells with siRNA-mediated downregulation of LAT2 and OLR1 exhibited enhanced gemcitabine sensitivity in MIAPaCa-2/GR and Rv-AsPC-1-GSTM3TV2 cells as determined using the CCK-8 assay and flow cytometric analysis (Fig. 7e–h). Moreover, LAT2 and OLR1 expression was abolished upon ectopic expression of let-7 in MIAPaCa-2/GR and Rv-AsPC-1-GSTM3TV2 cells (Fig. 7i, j). Thus, all these results confirmed the critical role of GSTM3TV2 in gemcitabine resistance of pancreatic cancer cells via LAT2 and OLR1.

### GSTM3TV2 is highly expressed in pancreatic cancer and associated with poor prognosis

**Fig. 8:**
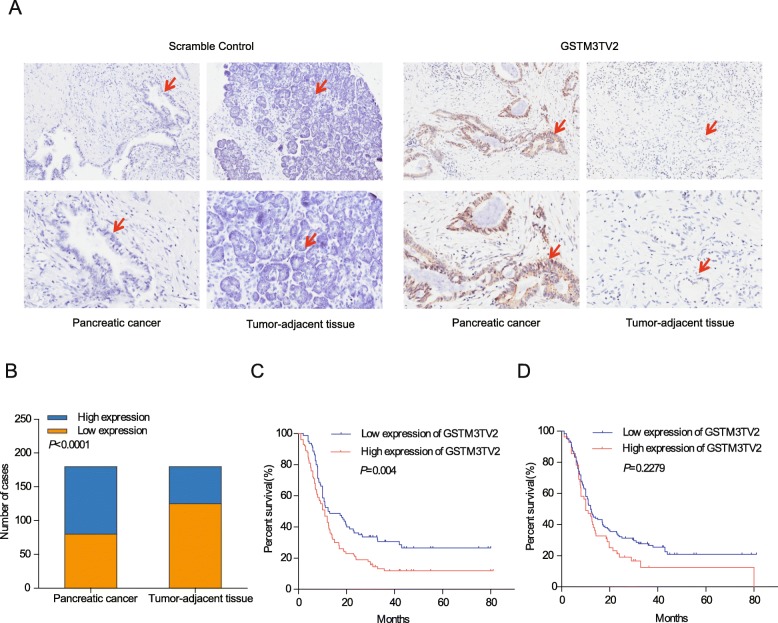
Clinical significance of GSTM3TV2 expression in patients with pancreatic cancer. **a** GSTM3TV2 expression in pancreatic cancer and tumour-adjacent tissues was analysed by ISH. **b** The expression levels of GSTM3TV2 were significantly increased in 180 pancreatic cancer tissue samples compared with those matched tumour-adjacent tissues as analysed using the Pearson *χ*^2^ test. **c** Kaplan-Meier survival analysis revealed that high levels of GSTM3TV2 expression in tumours were significantly associated with reduced survival in patients with pancreatic cancer. **d** High levels of GSTM3TV2 expression in tumour-adjacent tissues were not significantly associated with overall survival in patients

To explore whether GSTM3TV2 expression is associated with the poor prognosis of pancreatic cancer, we used ISH to measure the GSTM3TV2 expression levels in 180 pancreatic cancer tissue samples and matched tumour-adjacent tissues. The clinicopathological characteristics of the 180 patients are summarized in Additional file 7: Table S1. ISH staining revealed that GSTM3TV2 also localized to the cytoplasm in tissue cells (Fig. 8a). Among the 180 pancreatic cancer samples, 100 showed high-level GSTM3TV2 expression, and 80 showed low-level GSTM3TV2 expression. By contrast, 55 of 180 tumour-adjacent samples showed high-level GSTM3TV2 expression, and 125 showed low-level GSTM3TV2 expression. Thus, GSTM3TV2 levels were increased significantly in cancer tissues compared with those in tumour-adjacent tissues (Fig. 8b).

We next assessed the correlation between GSTM3TV2 levels and clinicopathological parameters and prognosis. We observed that GSTM3TV2 expression was significantly associated with lymph node staging and TNM staging but was not correlated with sex, age, tumour location, degree of differentiation, tumour staging and diabetes or perineural invasion (Additional file 7: Table S1). Univariate survival analysis indicated that differential degree, tumour staging, lymph node staging, TNM staging and GSTM3TV2 expression levels were potential prognostic factors of pancreatic cancer (Additional file 8: Table S2). Patients with high levels of GSTM3TV2 expression in tumour samples had significantly shorter overall survival (OS) than those with low levels of GSTM3TV2 expression (Fig. 8c). By contrast, there was no significant correlation between GSTM3TV2 expression in tumour-adjacent samples and OS (Fig. 8d). Notably, GSTM3TV2 expression in tumour samples was significantly correlated with OS in the following patient subgroups: late tumour staging, no lymph node metastasis, no perineural invasion, age < 65 years and no diabetes (Additional file 9: Figure S3A–E). Multivariable Cox regression analysis revealed that the degree of differentiation, TNM staging and elevated GSTM3TV2 expression were risk factors for OS in patients with pancreatic cancer (*P* = 0.008, hazard ratio [HR] = 1.706, 95% confidence interval [CI] 1.147–2.537; *P* = 0.003, HR = 2.786, 95% CI 1.149–5.470; *P* = 0.036, HR = 1.466, 95% CI 1.025–2.096, respectively) (Additional file 10: Table S3). Thus, these results indicated that GSTM3TV2 expression could be an independent risk factor for predicting OS.

## Discussion

Pancreatic cancer is a fatal disease; however, the emergence of drug resistance to gemcitabine or other therapeutic regimens contribute to this poor prognosis. Thus, investigating the mechanisms of drug resistance and re-sensitizing pancreatic cancer cells to drugs are important strategies for improving over survival. In the present study, we constructed a chemoresistance-related lncRNA-associated ceRNA network of pancreatic cancer and demonstrated lncRNA GSTM3TV2 acted as a key regulator of chemoresistance in pancreatic cancer. We found that GSTM3TV2 promoted pancreatic cancer gemcitabine resistance by upregulating LAT2 and OLR1 though competitively sponging let-7. In addition, we detected the GSTM3TV2 expression was significantly upregulated in pancreatic cancer tissues, and high expression of GSTM3TV2 had a worse prognosis. In this regard, our data contribute additional mechanisms to the elucidation of the molecular basis of chemoresistance in pancreatic cancer.

Previous studies have demonstrated that lncRNAs are involved in gemcitabine chemoresistance of pancreatic cancer. However, most studies only focused on specific lncRNAs, such as PVT1 [25–27], HOTTIP [28], HOTAIR [29] and TUG1 [30]. Only Zhou et al. [31] and Li et al. [32] screened for lncRNAs associated with chemoresistance of pancreatic cancer in gemcitabine-resistant SW1990/GZ cells via microarray analysis. To better define the lncRNAs associated with gemcitabine resistance, we screened the profile of lncRNAs associated with drug resistance and identified novel lncRNAs that were dysregulated in gemcitabine-resistant AsPC-1/GR cells. lncRNAs, including AFAP1-AS1 [33] and UCA1 [34], were reported to be overexpressed and act as oncogenes in the tumourigenesis of pancreatic cancer; however, these lncRNAs were also upregulated in gemcitabine-resistant AsPC-1/GR cell lines, which indicated that AFAP1-AS1 and UCA1 might be involved in the development of chemoresistance in pancreatic cancer. *Homo sapiens* glutathione S-transferase mu 3 transcript variant 2 (GSTM3TV2) is encoded from chromosome 1p13.3 and lacks an alternate exon in the 5′ coding region, which results in a frame shift and early stop codon and the significantly truncated transcription. Therefore, the predicted protein GSTM3 was not represented [provided by RefSeq, Nov. 2008]. Surprisingly, the lncRNA GSTM3TV2, which was identified in the present study, was not reported by Zhou et al. and Li et al. Possible explanations include the use of different cell lines in the experimental analyses and the different selection criteria between these studies.

Recently, the ceRNA hypothesis has been proposed to represent a novel posttranscriptional layer of gene regulation by acting as competitors for miRNAs [35]. It has been suggested that lncRNA-associated ceRNA crosstalk likely shifts under specific conditions and occurs in a disease-specific manner [36, 37]. Therefore, it is critical to study the functional roles and regulatory mechanisms of lncRNAs as ceRNAs in the chemoresistance of pancreatic cancer. In the present study, based on the ceRNA hypothesis, we constructed a specific lncRNA-associated ceRNA network focused on pancreatic cancer chemoresistance by utilizing associated miRNA, lncRNA and mRNA expression profiles between gemcitabine-resistant AsPC-1/GR cells and parental AsPC-1 cells. To our best knowledge, the specific chemoresistance-related lncRNA-associated ceRNA network in pancreatic cancer has not previously been reported in the literature. It has provided important clues for understanding the key roles of lncRNA-mediated gene regulation regarding chemoresistance in pancreatic cancer. At the same time, several lncRNAs (e.g., linc-ROR [15], GAS5 [16, 17] and linc-DYNC2H1-4 [18]) have been identified as ceRNAs to confer drug resistance in pancreatic cancer. In this study, we demonstrated that GSTM3TV2 acted as a key ceRNA to decrease gemcitabine-induced cytotoxicity in vitro and in vivo, which provided additional evidence for understanding lncRNA in chemoresistance of pancreatic cancer.

In addition, we next validated the role of the GSTM3TV2-associated ceRNA network on regulating chemoresistance in pancreatic cancer based on the constructed bioinformatics approach. Our findings indicated that GSTM3TV2 and LAT2/OLR1 physically associated with let-7 and functioned as ceRNAs. LAT2 is a member of the L-type amino acid transporter family, and its oncogenic role in the chemoresistance of pancreatic cancer has been recently reported by our group previous research [38]. OLR1 is also overexpressed in human cancers and has been found to participate in cancer cell proliferation, apoptosis, migration and angiogenesis [39–41]. Additionally, several lncRNAs (e.g., H19 [42], CCAT1 [43] and CCR492 [44]) have been reported to be ceRNA by sponging let-7 miRNA family in different types of cancer. We observed that LAT2 and OLR1 were upregulated in gemcitabine-resistant pancreatic cell lines and that inhibiting their expression enhanced the chemosensitivity of pancreatic cancer cells to gemcitabine. Consistently, LAT2 and OLR1 were direct targets of let-7 families, and GSTM3TV2 could upregulate the expression of LAT2/OLR1 in pancreatic cancer cells via competitively sponging let-7. Meanwhile, GSTM3TV2-mediated chemoresistance could be depressed by knocking down LAT2 and OLR1. Moreover, we also investigated the role of GSTM3 in pancreatic cancer cells using loss- and gain-of-function strategies, despite the fact that GSTM3TV2 does not affect GSTM3 expression. Our data revealed that GSTM3 did not significantly affect gemcitabine sensitivity of pancreatic cancer in vitro and in vivo, which implied that the oncogenic role of GSTM3TV2 in pancreatic cancer did not involve crosstalk with GSTM3 (Additional file 11: Figure S4). Thus, these results implied that GSTM3TV2 functioned as a molecular sponge for let-7 and upregulated the expression of its endogenous targets LAT2 and OLR1 to promote chemoresistance in pancreatic cancer.

Additionally, we also demonstrated that GSTM3TV2 expression was significantly increased in pancreatic cancer tissues and discovered a significant association between GSTM3TV2 expression and lymph node staging and TNM stage. Univariate and multivariable analyses revealed that GSTM3TV2 expression was an independent prognostic factor of pancreatic cancer, suggesting that GSTM3TV2 might be a useful prognostic biomarker to identify patients at increased risk of pancreatic cancer progression.

## Conclusions

Taken together, our results indicate that GSTM3TV2 is a valuable prognostic predictor of pancreatic cancer and appears to be a promising target for pancreatic cancer therapy.

## Additional files

Additional file 1: Supplemental Information. Lists of primer sequences, primary antibodies used and sequences for siRNA. (DOCX 26 kb)

Additional file 2: Supplementary Material 1. Upregulated lncRNAs and 385 downregulated lncRNAs. (XLS 577 kb)

Additional file 3: **Figure S1.** The hierarchical clustering of dysregulated mRNA (A) and microRNA (B) expression profiling among AsPC-1/GR and AsPC-1 cells. (PDF 1129 kb)

Additional file 4: Supplementary Material 2. mRNAs displayed differential expression. (XLS 3137 kb)

Additional file 5: Supplementary Material 3. miRNAs (> 2.0-fold change) displayed differential expression. (XLSX 74 kb)

Additional file 6: **Figure S2.** GSTM3TV2 enhances gemcitabine resistance of pancreatic cells. (A) qRT-PCR validation of GSTM3TV2 in pancreatic cancer cells. (B, C) Effects of GSTM3TV2 overexpression in MIAPaCa-2 cells on gemcitabine-induced cell death in pancreatic cells as determined using cell viability (B) and apoptosis assays (C). (D, E) Effects of GSTM3TV2 knockdown in AsPC-1/GR cells on gemcitabine-induced cell death in pancreatic cells as determined using cell viability (D) and apoptosis assays (E). (F) qRT-PCR analysis of GSTM3TV2 expression in Rv-AsPC-1-GSTM3TV2 and Rv-AsPC-1-NC cells. (G, H) Western blot analysis of LAT2, OLR1 in MIAPaCa-2/GR and Rv-AsPC-1-GSTM3TV2 cells. The data are presented as the mean ± SD. (Student’s t-test; *, *P* < 0.05). (PDF 777 kb)

Additional file 7: **Table S1.** Correlations of Lnc GSTM3TV2 levels in tissues and clinicopathological parameters. (XLSX 10 kb)

Additional file 8: **Table S2.** Univariate analyses of factors predictive of poor overall survival in pancreatic cancer patients. (XLSX 11 kb)

Additional file 9: **Figure S3.** Clinical significance of GSTM3TV2 expression in patients with pancreatic cancer (A-E) Subgroup analysis indicated that high levels of GSTM3TV2 expression significantly correlated with OS in patients with late tumour staging (T3+T4) (A), no lymph node metastasis (B), no perineuronal invasion (C), < 65 years of age (D), and without diabetes (E). (PDF 301 kb)

Additional file 10: **Table S3.** Multivariable analyses of factors predictive of poor overall survival in pancreatic cancer patients. (XLSX 10 kb)

Additional file 11: **Figure S4.** GSTM3 has no significant influence on gemcitabine sensitivity. (A) Effects of GSTM3 overexpression (A) or knockdown (C) on gemcitabine-induced cell death in pancreatic cells. (B, D) Effects of GSTM3TV2 overexpression (B) or knockdown (D) on cell apoptosis when incubated with or without gemcitabine in pancreatic cells. (E) Photographs of xenograft tumours developed from Lv-AsPC-1-GSTM3 and Lv-AsPC-1-NC cells in mice treated with gemcitabine. (F) The tumour volume and tumour weight of Lv-AsPC-1-GSTM3 and Lv-AsPC-1-NC when treated with gemcitabine in vivo. The data are presented as the mean ± SD (Student’s t-test; *, *P* < 0.05). (PDF 3245 kb)

## Data Availability

Not applicable.

## Abbreviations

ceRNA: Competing endogenous RNA
FDR: False discovery rate
GAPDH: Glyceraldehyde-3-phosphate dehydrogenase
GSTM3TV2: Glutathione S-transferase mu 3, transcript variant 2, noncoding RNA
LAT2: L-type amino acid transporter 2
lncRNA: The long noncoding RNA
OLR1: Oxidized low-density lipoprotein receptor 1
