# Supplementary material for: Long noncoding RNA GSTM3TV2 upregulates LAT2 and OLR1 by competitively sponging let-7 to promote gemcitabine resistance in pancreatic cancer
Source: J Hematol Oncol. 2019 Sep 12;12:97. doi: 10.1186/s13045-019-0777-7 (PMC6739963; doi:10.1186/s13045-019-0777-7)
Supplement: Supplementary file 1 — Supplemental Information. Lists of primer sequences, primary antibodies used and sequences for siRNA. (DOCX 26 kb) [file 13045_2019_777_MOESM1_ESM.docx]

**Supplemental Information**

**Figure S1.**The hierarchical clustering of dysregulated mRNA (A) and microRNA (B) expression profiling among AsPC-1/GR and AsPC-1 cells.

**Figure S2. GSTM3TV2 enhances gemcitabine resistance of pancreatic cells.** (A) qRT-PCR validation of the efficacy of GSTM3TV2 overexpression and knockdown in pancreatic cancer cells. (B, C) Effects of GSTM3TV2 overexpression in MIAPaCa-2 cells on gemcitabine-induced cell death in pancreatic cells as determined using cell viability (B) and apoptosis assays (C). (D, E) Effects of GSTM3TV2 knockdown in AsPC-1/GR cells on gemcitabine-induced cell death in pancreatic cells as determined using cell viability (D) and apoptosis assays (E). The data are presented as the mean ± SD (*, P<0.05). (F)qRT-PCR analysis of GSTM3TV2 expression in Rv-AsPC-1-GSTM3TV2 and Rv-AsPC-1-NC cells. (G, H) Western blot analysis shown that the expression levels of LAT2, OLR1 were downregulated when LAT2, OLR1knockdown in MIAPaCa-2/GR and Rv-AsPC-1-GSTM3TV2 cells. The data are presented as the mean ± SD. (Student’s t-test; *, P < 0.05).

**Figure S3. Clinical significance of GSTM3TV2 expression in patients with pancreatic cancer** (A-E) Subgroup analysis indicated that high levels of GSTM3TV2 expression significantly correlated with OS in patients with late tumour staging (T3+T4) (A), no lymph node metastasis (B), no perineuronal invasion (C), < 65 years of age (D), and without diabetes (E).

**Figure S4. GSTM3 has no significant influence on gemcitabine sensitivity.**

(A) Effects of GSTM3 overexpression (A) or knockdown (C) on gemcitabine-induced cell death in pancreatic cells as determined by detecting cell viability. (B, D) Effects of GSTM3TV2 overexpression (B) or knockdown (D) on cell apoptosis when incubated with or without gemcitabine in pancreatic cells as determined by detecting apoptosis rates. (E) Photographs of xenograft tumours developed from Lv-AsPC-1-GSTM3 and Lv-AsPC-1-NC cells in mice treated with gemcitabine. (F) The chemotherapeutic sensitivities of the pancreatic cancer cell lines Lv-AsPC-1-GSTM3 and Lv-AsPC-1-NC to gemcitabine were examined in vivo by measuring the tumour volume and tumour weight. The data are presented as the mean ± SD (Student’s t-test; *, P < 0.05).

**Methods**

**Microarray analysis**

Total RNA was extracted from AsPC-1 and AsPC-1/GR cells using TRIzol RNA isolation reagent (Invitrogen, Carlsbad, CA, USA) according to the manufacturer’s protocol. RNA quality and quantity were assessed using capillary electrophoresis with Fragment Analyzer and Standard/High Sensitivity RNA Analysis kits (Advanced Analytical Technologies, Ames, IA, U.S.)

To identify the miRNA profiles associated with chemoresistance of pancreatic cancer, an Affymetrix GeneChip miRNA Array v. 4.0 (Affymetrix, Santa Clara, CA, U.S.) was used according to the manufacturer's protocol. RNA samples (1 µg) were labelled with a FlashTag Biotin RNA Labeling Kit (Genisphere, Hatfield, PA). The labelled RNA was then washed and stained using an Affymetrix GeneChip Hybridization Wash and Stain Kit and scanned using an Affymetrix GeneChip Scanner 3000 7G (Affymetrix, Santa Clara, CA, U.S.). The data were analysed using Affymetrix GeneChip Command Console software (Affymetrix). Fold change either > 2 or < 0.5 and P < 0.05 (Student t tests) were applied for selecting miRNAs specific to drug-resistance.

To identify the mRNA profiles associated with chemoresistance of pancreatic cancer, an Affymetrix GeneChip Human Transcriptome Array 2.0 (Affymetrix) was used according to the manufacturer's protocol. Biotinylated complementary DNA (cDNA) was prepared from 500 ng of total RNA. After labelling and hybridizing, the GeneChips were washed and stained using an Affymetrix Fluidics Station 450 and then scanned with an Affymetrix GeneChip Scanner 3000 7G. The data were analysed with a Robust Multichip Analysis algorithm using the Affymetrix default analysis settings with global scaling as the normalization method. To determine the significance of the differences and the false discovery rate (FDR), thresholds of P < 0.05 and FDR < 0.05 were used. Gene expression fold changed either >1.5 or < 0.66 were set as the default filter criteria for identifying significant differentially expressed genes.

**Bioinformatics method**

To construct chemoresistance-related lncRNA-associated ceRNA network, we focused on miRNAs, mRNAs and lncRNAs that were significantly downregulated or upregulated (P < 0.05). According to our previous quantitative study of ceRNA effects, the competing effects are more pronounced for miRNAs with moderate expression level, but not the lowly or most highly expressed miRNAs. In addition, the ceRNA mediated de-repression effect depends on the expression level of target genes and binding affinities between miRNA and targets. Thus we focused on appropriately expressed miRNAs with an expression intensities greater than 2048 but less than 11585 and mRNAs and lncRNAs with relatively high expression level and significant fold changes with an expression intensity greater than 500 and fold change greater than 1.75 -fold. Then, we mapped the sequences of the selected miRNAs, mRNAs and lncRNAs. Using the TargetScan tool^1 2^, we calculated the binding score among the selected miRNAs with the selected mRNAs and lncRNAs as the initial ceRNA candidates. We then filtered and selected matches with a score lower than 0, which indicate that stronger predictive values of the binding sites and calculated binding site numbers among the selected miRNAs with the mRNAs and lncRNAs. Finally, we evaluated the potential ceRNAs between two mRNAs or lncRNAs by calculating the total number of binding sites among their target competitive miRNAs (with a same direction of expression changes between the two mRNAs or lncRNAs) and then selected paired which have more competitive sites (Figure2B showed the result which have more than 2 competitive sites) as predicted candidate ceRNA pairs.

**The primers sequences are listed below.**

| Gene | Forward Primer (5' - 3') | Reverse Primer (5' - 3') |
| --- | --- | --- |
| GSTM3TV2 | CTAGTCCTCGAAGGCTCGGA | TCATAGTCAGGAGCCCACGA |
| GSTM3 | TCCTGGAGTTCACGGATACCT | GCCATTGGCTTCGATCATAGTC |
| EGFR | CAAGGCACGAGTAACAAG | GGCAATGAGGACATAACC |
| hsa-let-7g-5p | GTCGTATCCAGTGCAGGGTCCGAGGTATTCGCACTGGATACGACAACTGTAC | TCGCGCTGAGGTAGTAGTT |
| hsa-let-7f-5p | GTCGTATCCAGTGCAGGGTCCGAGGTATTCGCACTGGATACGACAACTATAC | TCGCGCTGAGGTAGTAGAT |
| hsa-let-7d-5p | GTCGTATCCAGTGCAGGGTCCGAGGTATTCGCACTGGATACGACAACTATGC | TCGCGCAGAGGTAGTAGGT |
| GADPH | CGGAGTCAACGGATTTGGTCGTAT | AGCCTTCTCCATGGTGGTGAAGAC |
| PARM1 | GTACAGAGTCTGCCTACATCAGC | TGGAGAGCTAGTCCAGATGGT |
| LAT2 | AGGCTGGAACTTTCTGAATTACG | ACATAAGCGACATTGGCAAAGA |
| OLR1 | TTGCCTGGGATTAGTAGTGACC | GCTTGCTCTTGTGTTAGGAGGT |
| GLO1 | AGCAGACCATGCTACGAGTGA | GAGAGCGCCCAGGCTATTT |
| KLK6 | CCAAACTCTCTGAACTCATCCAG | GTGTCAGGGAAATCACCATCTG |

**Primary antibodies used are listed below.**

| c-Myc | Cell Signaling Technology | 13987S |
| --- | --- | --- |
| HMGA2 | Cell Signaling Technology | 8179S |
| OLR1 | Abcam | ab126538 |
| LAT2 | Abcam | ab123896 |
| Ras | Cell Signaling Technology | 3965S |
| β-actin | Cell Signaling Technology | 4970 |

**The sequences for the siRNA are listed below**

| siRNA | Forward Primer (5' - 3') |
| --- | --- |
| siGSTM3TV2 | CGTGGGCTCCTGACTATGA |
| siGSTM3 | TATGAGGAGAAACGGTACA |

**Supplemental References**

1. Agarwal V, Bell GW, Nam JW, et al. Predicting effective microRNA target sites in mammalian mRNAs. *eLife* 2015;4 doi: 10.7554/eLife.05005 [published Online First: 2015/08/13]

2. Yuan Y, Liu B, Xie P, et al. Model-guided quantitative analysis of microRNA-mediated regulation on competing endogenous RNAs using a synthetic gene circuit. *Proceedings of the National Academy of Sciences of the United States of America* 2015;112(10):3158-63. doi: 10.1073/pnas.1413896112 [published Online First: 2015/02/26]
