# Supplementary figures and images for: Long noncoding RNA GSTM3TV2 upregulates LAT2 and OLR1 by competitively sponging let-7 to promote gemcitabine resistance in pancreatic cancer
Source: J Hematol Oncol. 2019 Sep 12;12:97. doi: 10.1186/s13045-019-0777-7 (PMC6739963; doi:10.1186/s13045-019-0777-7)

Figure S1

A

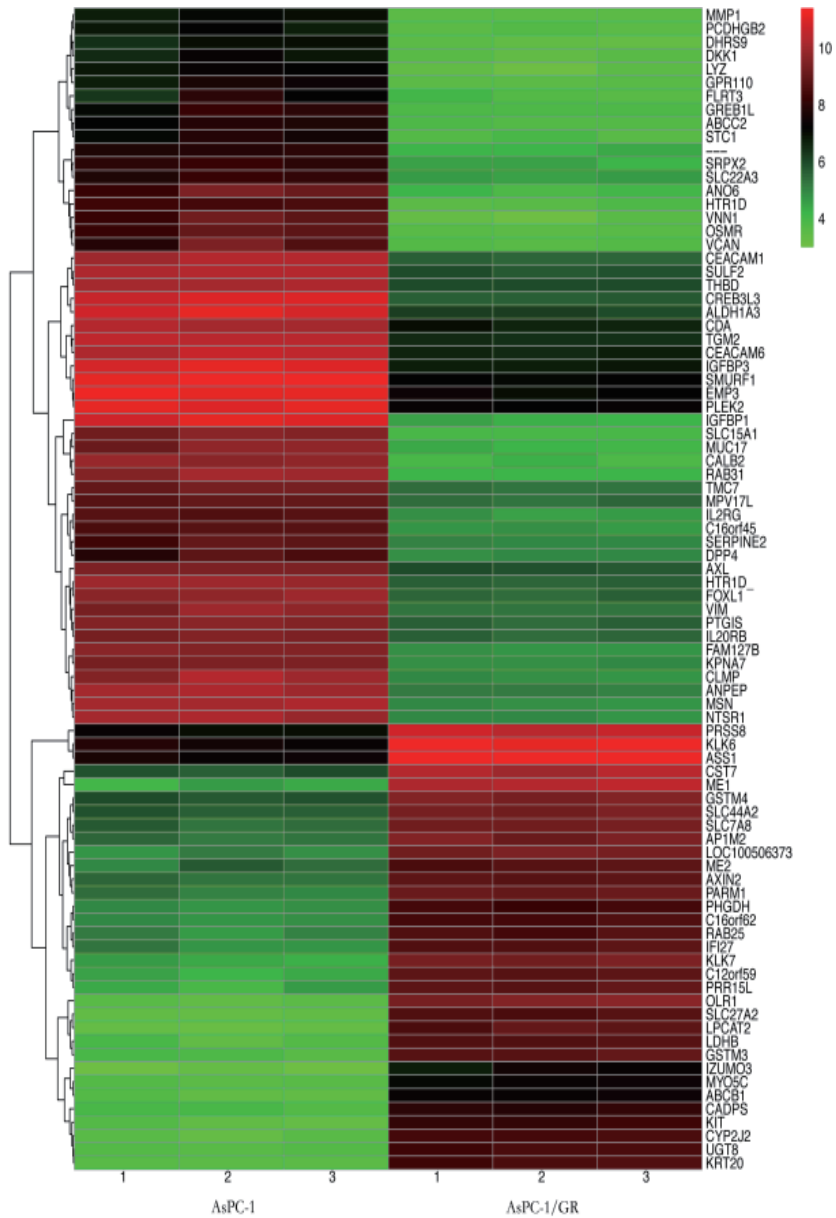

B

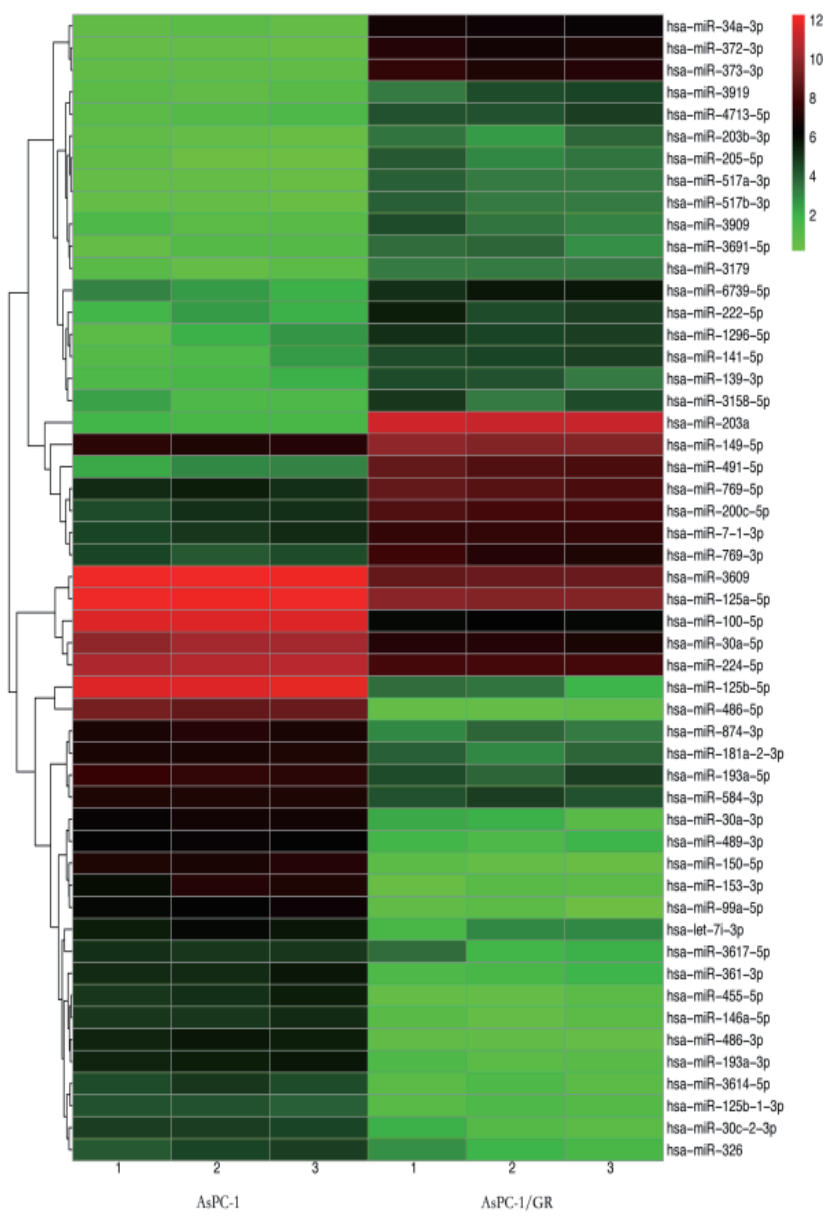

Supplement: Supplementary file 3 — Figure S1. The hierarchical clustering of dysregulated mRNA (A) and microRNA (B) expression profiling among AsPC-1/GR and AsPC-1 cells. (PDF 1129 kb) [file 13045_2019_777_MOESM3_ESM.pdf]

Figure S2

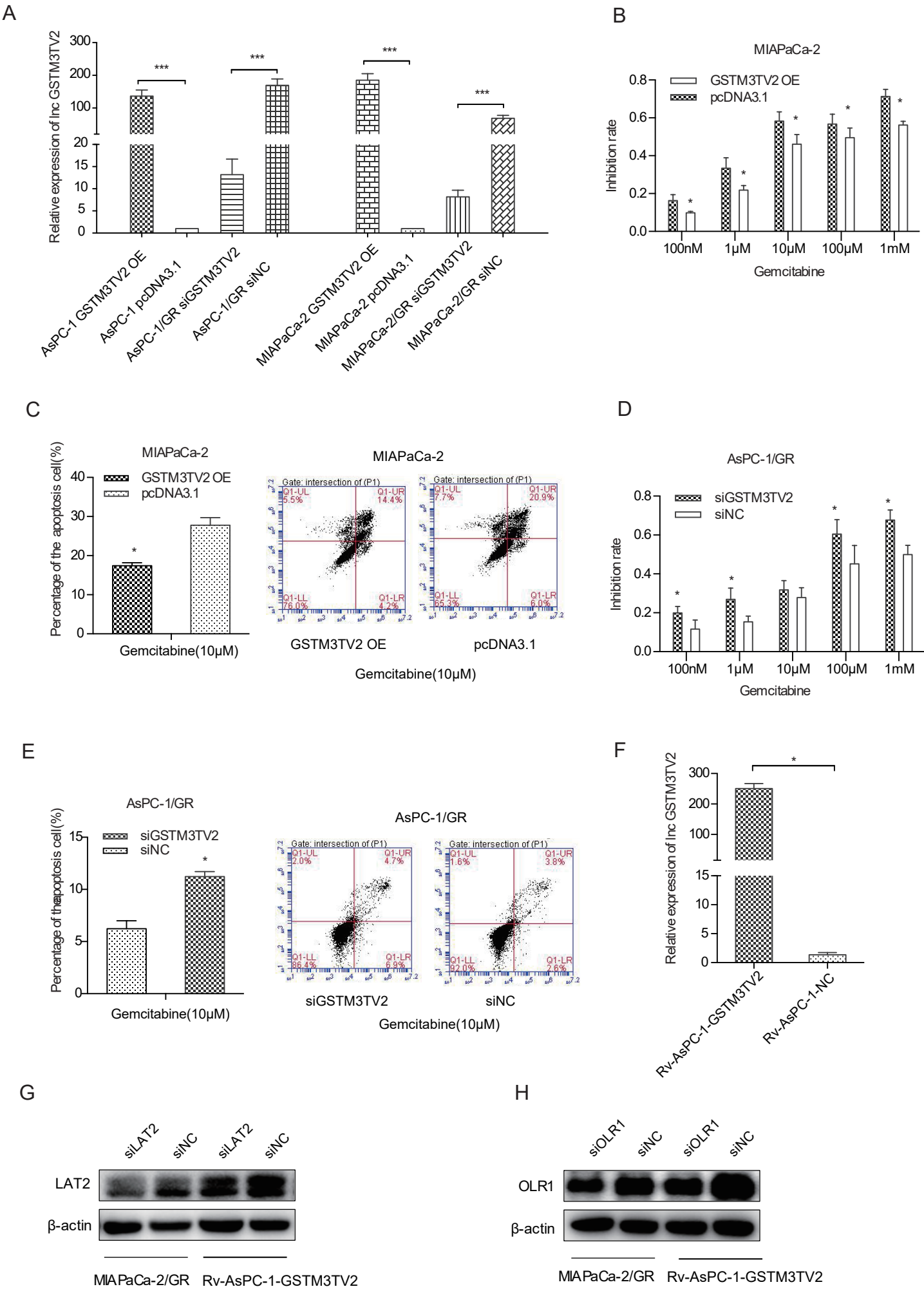

Supplement: Supplementary file 6 — Figure S2. GSTM3TV2 enhances gemcitabine resistance of pancreatic cells. (A) qRT-PCR validation of GSTM3TV2 in pancreatic cancer cells. (B, C) Effects of GSTM3TV2 overexpression in MIAPaCa-2 cells on gemcitabine-induced cell death in pancreatic cells as determined using cell viability (B) and apoptosis assays (C). (D, E) Effects of GSTM3TV2 knockdown in AsPC-1/GR cells on gemcitabine-induced cell death in pancreatic cells as determined using cell viability (D) and apoptosis assays (E). (F) qRT-PCR analysis of GSTM3TV2 expression in Rv-AsPC-1-GSTM3TV2 and Rv-AsPC-1-NC cells. (G, H) Western blot analysis of LAT2, OLR1 in MIAPaCa-2/GR and Rv-AsPC-1-GSTM3TV2 cells. The data are presented as the mean ± SD. (Student’s t-test; *, P < 0.05). (PDF 777 kb) [file 13045_2019_777_MOESM6_ESM.pdf]

Figure S3

A

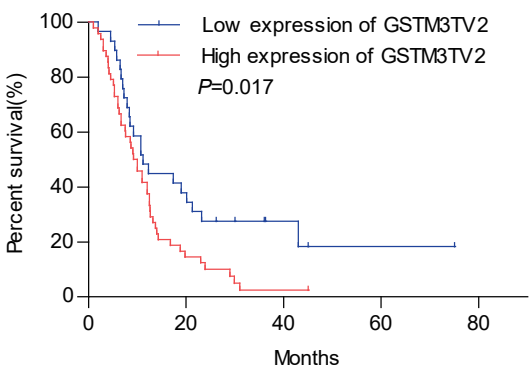

B

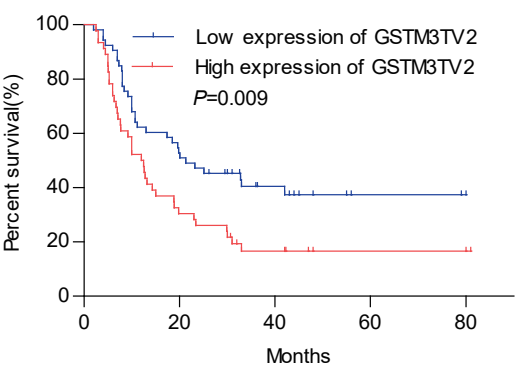

C

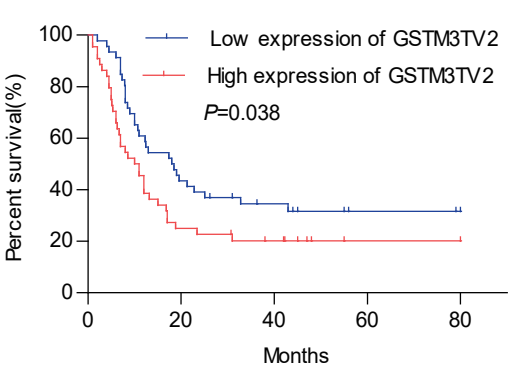

D

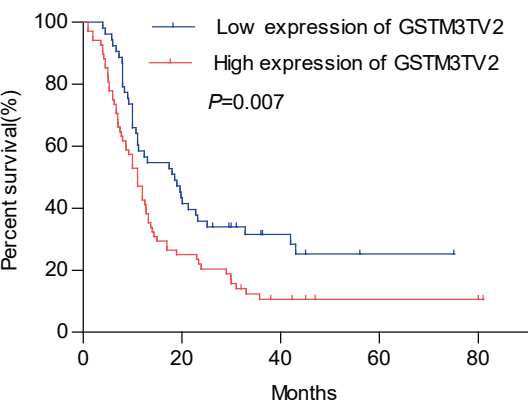

E

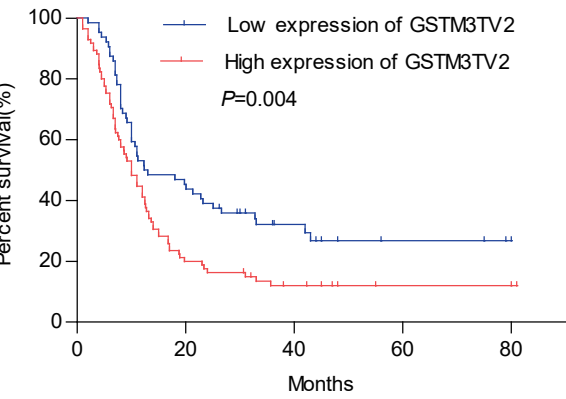

Supplement: Supplementary file 9 — Figure S3. Clinical significance of GSTM3TV2 expression in patients with pancreatic cancer (A-E) Subgroup analysis indicated that high levels of GSTM3TV2 expression significantly correlated with OS in patients with late tumour staging (T3+T4) (A), no lymph node metastasis (B), no perineuronal invasion (C), < 65 years of age (D), and without diabetes (E). (PDF 301 kb) [file 13045_2019_777_MOESM9_ESM.pdf]

Figure S4

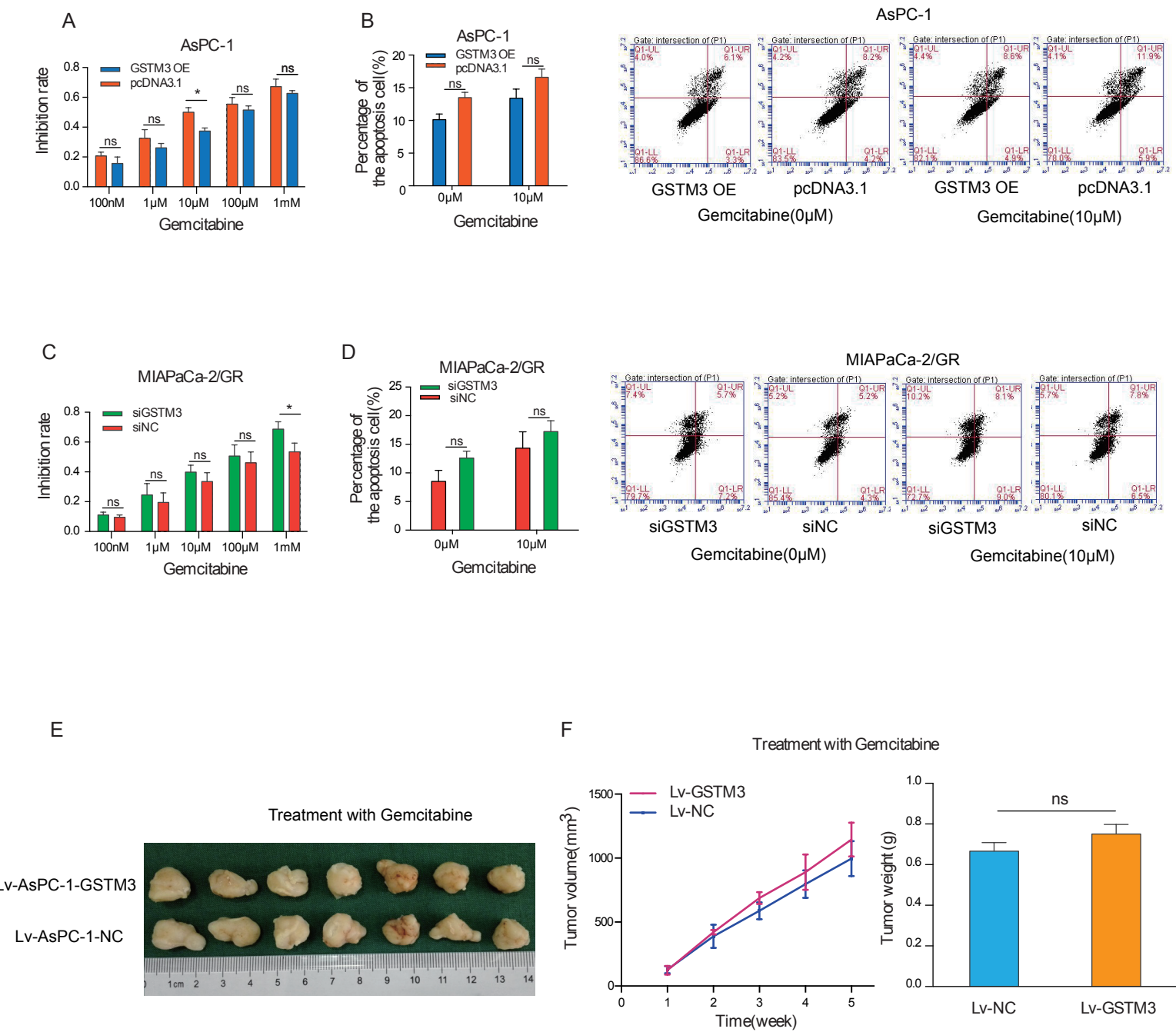

Supplement: Supplementary file 11 — Figure S4. GSTM3 has no significant influence on gemcitabine sensitivity. (A) Effects of GSTM3 overexpression (A) or knockdown (C) on gemcitabine-induced cell death in pancreatic cells. (B, D) Effects of GSTM3TV2 overexpression (B) or knockdown (D) on cell apoptosis when incubated with or without gemcitabine in pancreatic cells. (E) Photographs of xenograft tumours developed from Lv-AsPC-1-GSTM3 and Lv-AsPC-1-NC cells in mice treated with gemcitabine. (F) The tumour volume and tumour weight of Lv-AsPC-1-GSTM3 and Lv-AsPC-1-NC when treated with gemcitabine in vivo. The data are presented as the mean ± SD (Student’s t-test; *, P < 0.05). (PDF 3245 kb) [file 13045_2019_777_MOESM11_ESM.pdf]
